# TOPAZ1, a Novel Germ Cell-Specific Expressed Gene Conserved during Evolution across Vertebrates

**DOI:** 10.1371/journal.pone.0026950

**Published:** 2011-11-01

**Authors:** Adrienne Baillet, Ronan Le Bouffant, Jean Nicolas Volff, Alix Luangpraseuth, Elodie Poumerol, Dominique Thépot, Eric Pailhoux, Gabriel Livera, Corinne Cotinot, Béatrice Mandon-Pépin

**Affiliations:** 1 INRA, UMR 1198 Biologie du Développement et Reproduction, Jouy-en-Josas, France; 2 ENVA, Maisons Alfort, France; 3 CEA, DSV/DRR/SEGG/LDRG, Laboratory of Differentiation and Radiobiology of the Gonads, Unit of Gametogenesis and Genotoxicity, Fontenay aux Roses, France; 4 Institut de Génomique Fonctionnelle de Lyon, Université de Lyon, CNRS, INRA, Ecole Normale Supérieure de Lyon, Lyon, France; Stanford University, United States of America

## Abstract

**Background:**

We had previously reported that the Suppression Subtractive Hybridization (SSH) approach was relevant for the isolation of new mammalian genes involved in oogenesis and early follicle development. Some of these transcripts might be potential new oocyte and granulosa cell markers. We have now characterized one of them, named *TOPAZ1* for the Testis and Ovary-specific PAZ domain gene.

**Principal Findings:**

Sheep and mouse *TOPAZ1* mRNA have 4,803 bp and 4,962 bp open reading frames (20 exons), respectively, and encode putative TOPAZ1 proteins containing 1,600 and 1653 amino acids. They possess PAZ and CCCH domains. In sheep, *TOPAZ1* mRNA is preferentially expressed in females during fetal life with a peak during prophase I of meiosis, and in males during adulthood. In the mouse, *Topaz1* is a germ cell-specific gene. TOPAZ1 protein is highly conserved in vertebrates and specifically expressed in mouse and sheep gonads. It is localized in the cytoplasm of germ cells from the sheep fetal ovary and mouse adult testis.

**Conclusions:**

We have identified a novel PAZ-domain protein that is abundantly expressed in the gonads during germ cell meiosis. The expression pattern of *TOPAZ1,* and its high degree of conservation, suggests that it may play an important role in germ cell development. Further characterization of *TOPAZ1* may elucidate the mechanisms involved in gametogenesis, and particularly in the RNA silencing process in the germ line.

## Introduction

Ovarian development in mammals involves coordinated and multiple and complex molecular, cellular and morphogenetic events. Unlike testis differentiation, where germ cells are dispensable, oocytes are required to induce and maintain ovarian somatic cells [1]. Some key events occur during development of the fetal mammalian ovary, including, colonization of the undifferentiated gonad by primordial germ cells (PGCs) and then their proliferation, the entrance of germ cells into meiosis, atresia of germ cells and the formation of primordial follicles [2]. Although animal germline development varies in different species, some general rules have emerged. During the earliest embryonic stages, PGCs proliferate in a niche supported by somatic cells and then differentiate into germline stem cells, which proliferate through self-renewal. Formation of the germ cell nest (or cyst) is an important step in germline differentiation that has been conserved during evolution from insects to vertebrates [3], [4]. Oogonia in nests are clonally derived [5] and germline nest formation occurs through incomplete cytokinesis during mitosis, resulting in the connection of daughter cells by intercellular bridges [6], [7]. In the mouse, the maximum number of germ cell clusters is detected on embryonic day 13.5, just prior to the commitment of PGCs to the oocyte developmental program and entry into meiosis [8]. Within germ cell nests, the oogonia enter meiotic prophase [9], [10]. Several global approaches using microarray technologies have been used to isolate the genes involved in meiosis by comparing transcriptional activity in ovaries with or without germ cells in meiotic prophase I [11]–[15]. Both germ cell-intrinsic and -extrinsic factors govern meiotic initiation in mouse embryos [16], [17]. The RNA-binding protein DAZL (Deleted in AZospermia-like), expressed by both male and female post-migratory germ cells, is one of the first intrinsic factors necessary to enroll germ cells in the meiosis event [17] in response to retinoic acid (RA). Entry into meiosis is then controlled by RA signals that are blocked in the testis by CYP26B1 (cytochrome P450 26B1) [18]–[20] and fibroblast growth factor 9 (FGF9) [21]. In the fetal ovary, RA signaling acts on germ cells to promote the expression of the cytoplasmic factor Stra8 (Stimulated by Retinoic acid). Stra8 is required for premeiotic DNA synthesis, meiotic initiation and meiotic progression in germ cells [22], [23]. Consistent with the central role that RA is believed to play in vertebrate germline development and the sex-specific timing of entry into meiosis, RA appears to function as one of the principal inducers of Stra8 expression in early germ cells [24]–[27]. However, another study reported that Stra 8 expression in the mouse fetal ovary does not require RA signaling, and that Stra8 expression is expressed in the absence of physiologically detectable levels of RA [28]. That study demonstrated that another unknown inducer of Stra8 might be involved in Stra8 expression to enable the initiation of mouse meiosis.

Just after birth in the mouse, and during fetal life in ruminants and humans, the nests break down to form individual primordial follicles [8], [29], [30]. Their formation, and the transition to primary follicles, represents a critical period of follicle formation [31], [32]. Ovarian follicles are the functional units within the female gonad that nurture oocyte maturation and enable the production of steroid hormones.

During the past ten years, many of the growth factors and signaling proteins that mediate the early stages of folliculogenesis have been identified using mouse genetic models, *in vivo* injection studies, and *ex vivo* organ culture techniques. These studies revealed important roles for the transforming growth factor beta (*Tgf-beta*) superfamily of proteins in the ovary [33]–[35]. Moreover, several transcription factors are known to affect the regulation of oocyte-specific genes during early folliculogenesis (**Figure S1**). *Figla* (a factor in the germline alpha) regulates the expression of numerous genes in the ovary, including *Zp2*, *Pou5f1*, *Nlrp14*, *Nlrp4f*, and *Nlrp4b* [36], [37]. *Nobox* (newborn ovary homeobox gene) is necessary for the expression of several key oocyte-specific genes, including *Gdf9* and *Pou5f1* [38]–[40]. *Sohlh1* (spermatogenesis- and oogenesis-specific bHLH transcription factor 1) is another germ cell-specific gene that encodes a bHLH protein [41]. *Sohlh1* lies upstream of *Lhx8*, *Zp1*, and *Zp3* and is preferentially expressed in primordial oocytes [41]. More recently, Rajkovic's team described a novel spermatogenesis- and oogenesis-specific basic helix-loop-helix (bHLH) transcription factor, *Sohlh2* [42], [43]. *Sohlh2*-deficient ovaries can form primordial follicles that do not differentiate surrounding granulosa cells into cuboidal and multilayered structures [43].

In addition to these functional relationships, small non-coding RNAs, including microRNAs (miRNAs), small interfering RNAs (siRNAs), and Piwi-interacting RNAs (piRNAs) have been implicated as key regulators, especially in the gonads of eukaryotes [44]. It has been shown that piRNAs become abundant in germ cells at around the pachytene stage of prophase of meiosis I [45], but they may be present at lower levels during earlier stages. It has been shown that piRNAs repress transposable elements (TEs) but their molecular functions are still unknown [46]–[49]. Unlike microRNAs, individual piRNAs sequences per se are not conserved.

In order to discover novel genes expressed in the ovary during prophase I meiosis of germ cells and follicle formation, we previously reported on the generation of two sheep fetal ovary cDNA libraries, obtained by applying SSH using mRNAs isolated from 55 and 82 day *post-coitum* (d*pc*) female gonads [50]. In this species, meiosis occurs between days 50 and 75 and the first primordial follicles are observed at around days 75–80 of gestation [51]. In the resulting libraries [50], we found several transcripts with sex or stage-specific patterns of expression, such as *MOV10L1* (EMBL:CU654655), [52]–[54], *TEX11* (EMBL:CU651916), [55] and Gametocyte-specific factor 1 (*GTSF1*) (EMBL:CU652509), [56]. Among these transcripts, we isolate an EST encoding a putative protein with a PAZ domain (Piwi/Argonaute/Zwille), namely *TOPAZ1* (EMBL: CU654960 and CU655260). During the current study, we characterize the genomic structure of the *TOPAZ1* gene and determine its expression pattern during development in sheep and mouse. We show here that *TOPAZ1/Topaz1* is expressed in both the male and female gonads and is highly conserved among vertebrates, including fish, amphibians, birds and mammals. Furthermore, we demonstrate that *TOPAZ1/Topaz1* mRNA and TOPAZ1 protein are localized in both male and female germ cells lineages.

## Results

### Characterization of *TOPAZ1* transcripts

**Figure 1:**
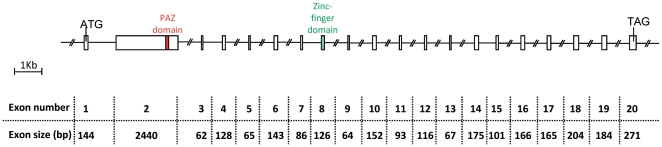
Structure of the sheep *TOPAZ1* gene. Sheep *TOPAZ1* cDNA derived from exons 1–20 and contained 4952 nucleotides. *TOPAZ1* was composed of twenty exons in sheep, bovine, human and mouse. The PAZ domain is located in the second exon and the CCCH domain in exon 8. The nucleotide number of each exon of sheep *TOPAZ1* is indicated under the Figure.

*CU654960 and CU655260 ESTs* arising from our previously described sheep fetal ovary libraries [50] were aligned with public EST databases and displayed significant similarity with human testis EST (Genbank AI208289): identities = 393/428 (92%); e value = 2e-171. As this sheep EST was expressed in the testis and ovary and contained a PAZ-domain, we decided to call it *TOPAZ1.* In order to characterize the full-length sequence of the *TOPAZ1* transcript in sheep, 5′RACE experiments were performed using mRNA from female fetal ovaries of 60 days *post-coitum* (60 d*pc*). The primers used during the 5′RACE experiments are listed in **Table S1**. Moreover, the rest of the coding sequence of *TOPAZ1* cDNA was cloned by successive PCR procedures from sheep fetal ovaries of 60 d*pc*. The primers used to clone sheep *TOPAZ1* cDNA are listed in **Table S2**. We found that the full length *TOPAZ1* cDNA sequence contained 20 exons with an open reading frame sequence of 4803 bp. The number of nucleotides for each exon is shown in **Figure 1**. The putative TOPAZ1 protein contained 1600 amino acids.

By searching for sequences with similarities to sheep *TOPAZ1* gene within public databases, we found the human *TOPAZ1* gene (Official Symbol in GeneBank C3orf77; GeneBank Accession number NM_001145030, Ensembl ENSG00000173769; UniGene Hs.132125; human hypothetical protein LOC375337). Full length human *TOPAZ1* cDNA (ORF: 5079 bp) was also made up of 20 exons, as in sheep. Moreover, similarity analysis highlighted the mouse *Topaz1* gene (Official Symbol in GeneBank Gm9524; GeneBank Accession number XM_001474362, UniGene Mm.461223; mouse hypothetical protein LOC100032115) but this predicted gene was much shorter (ORF: 1851 bp) when compared to sheep and human *TOPAZ1*. In order to verify the exact size of the mouse *Topaz1* cDNA, we designed oligonucleotide primers (**Table S3**) and ran successive RT-PCR using mouse adult testis mRNA. We were thus able to deduce the size of the mouse *Topaz1* cDNA ORF as being 4962 bp, which was much longer than the predicted sequence available in public databases (GeneBank Accession number XM_001474362: 1919 bp, ORF: 1851 bp).

In these three species of mammals, all exons had almost the same nucleotide size, except for exon 1 (see **Figure S2**). Indeed, in its 3′ part, the human *TOPAZ1* exon 1 contained 141 bp more than the sheep and mouse genes. Moreover, using RT-PCR and sequencing we showed that the location of the predicted start codon (ATG) differed between sheep and mouse. In the latter species, the beginning of the ORF is at the same position as the human protein start codon (UniProtKB: Q8N9V7).

**Figure 2:**
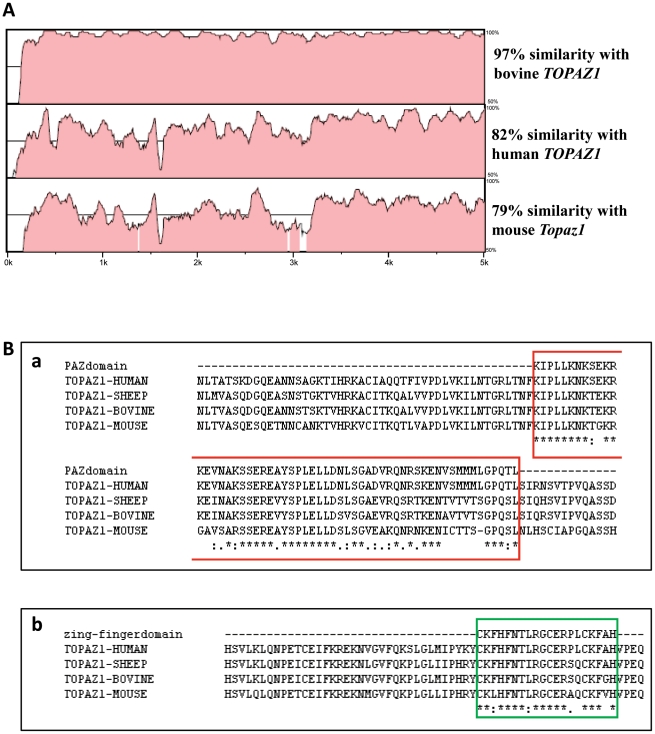
(A) Nucleotide sequence conservation of mammalian *TOPAZ1* transcripts and (B) Conservation of PAZ and CCCH domains of TOPAZ1 protein in mammalian species. (A) The percentage nucleotide identity between sheep *TOPAZ1* cDNA and that of other mammalian species is indicated on the right-hand side. These alignments were obtained using the Vista Genome browser (http://genome.lbl.gov/vista/index.shtml). (Ba) The PAZ domain surround (in red) consisted of 58 amino acids. The percentages of identity were 96%, 78%, and 71% between sheep-cattle, sheep-human and sheep-mouse, respectively. (Bb) The CCCH domain is indicated in the green box, and consisted of 20 amino acids. Amino acid identity was 90%, 85%, and 80% between sheep-cattle, sheep-human and sheep-mouse, respectively. Identical residues are noted with an asterisk.

According to the Vista Genome browser (http://genome.lbl.gov/vista/index.shtml), the nucleotide similarity between the ORF of the *TOPAZ1* mRNA of sheep and of different mammalian species (cattle: BK008402, human: NM_001145030 and mouse: HM631980) was high, ranging from 79% between sheep and mice and 82% between sheep and human to 97% between sheep and cattle (**Figure 2A**) and the highest degree of similarity across species existed from 3.25 to 5.0 Kb on the *TOPAZ1* cDNA sequences. *TOPAZ1* localized to human chromosome HS 3p21.33, its mouse orthologue to chromosome 9, and the cattle gene to chromosome 22.

We have submitted these sheep and mouse *TOPAZ/Topaz1* sequences to the GenBank databases and the accession numbers are: HM631979 (*Ovis aries*) and HM631980 (*Mus musculus*). We also submitted the predicted cattle TOPAZ1 cDNA (*Bos taurus*) to Genbank: nucleotide sequence data reported are available in the Third Party Annotation Section of the DDBJ/EMBL/GenBank databases under the accession number TPA: BK008402.

### TOPAZ1 protein contains conserved PAZ and zinc finger domains

#### PAZ domain

TOPAZ1 protein belongs to a functionally uncharacterized protein family (mouse hypothetical protein LOC671232; human hypothetical protein LOC375337) containing an evolutionarily conserved domain named PAZ for Piwi/Argonaut/Zwille (PF02170, Accession IPR003100) located in exon 2 (**Figure 1**) of all aligned sequences (amino acids 688 to 745 in human; 593 to 650 in ovine; 646 to 703 in bovine; 651 to 708 in mouse). This domain was found in two families of proteins that are involved in post-transcriptional gene silencing, namely the Piwi and Dicer families. The conservation of the PAZ domain in different species was high (between 71% and 96%) and is shown in **Figure 2** **(Ba)**.

#### Other domains

In addition, TOPAZ1 protein contains a conformation typical zinc-finger domain (amino acids 1097 to 1116 in humans; 1005 to 1024 in sheep (**Figure 1**); 1058 to 1077 in cattle and 1056 to 1075 in mouse) of C-x8-C-x5-C-x3-H type (PF00642, Accession IPR000571), but which is C-x9-C-x4/5-C-x3-H in the TOPAZ1 proteins of several species (**Figure 2 (Bb)**).

### TOPAZ1 protein is phylogenetically conserved in Vertebrates

**Figure 3:**
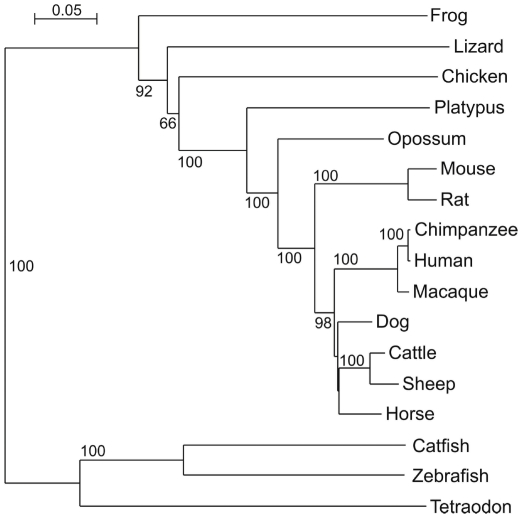
Molecular phylogeny of TOPAZ1. Phylogenetic analysis was performed on a 620 amino-acid alignment extending from the CCCH zinc finger domain to the C-terminal end using the neighbor-joining method [57]; (1,000 bootstrap replicates). The tree was not rooted. Accession numbers: *Homo sapiens* (human) NP_001138502; *Pan troglodytes* (chimpanzee) XP_526186; *Macaca mulatta* (rhesus monkey) XP_001114967; *Mus musculus* (house mouse) EDL09106; *Rattus norvegicus* (rat) EDL76795; *Bos taurus* (cattle) NW_001494083.2 (genomic); *Ovis aries* (sheep) HM631979; *Equus caballus* (horse) NW_001867381.1 (genomic); *Canis familiaris* (dog) NW_876276.1 (genomic); *Monodelphis domestica* (gray short-tailed opossum) XP_001381211; *Ornithorhynchus anatinus* (duck-billed platypus) NW_001794448.1 (genomic); *Gallus gallus* (chicken) NW_001471633.1 (genomic); *Anolis carolinensis* (green anole, lizard) ENSEMBL scaffold_98 (genomic); *Xenopus tropicalis* (frog) ENSEMBL scaffold_320 (genomic); *Ictalurus punctatus* (catfish) ABD91555; *Danio rerio* (zebrafish) NW_001877999.1 (genomic); *Tetraodon nigroviridis* (pufferfish) ENSEMBL Chr. 21.

To determine whether the TOPAZ1 protein has been conserved during evolution, a phylogenetic analysis was performed on a 620 amino-acid alignment extending from the CCCH zinc finger to the C-terminal end using the neighbor-joining method [57], (1,000 bootstrap replicates). We showed that TOPAZ1 proteins were phylogenetically very close between sheep and cattle, and neighbors with horse and dog orthologs (**Figure 3**). Moreover, these 620 TOPAZ1 amino acids were aligned within several classes of vertebrates such as fish, amphibians and birds, and displayed a high degree of conservation within the Vertebrate Subphylum. Thus TOPAZ1 proteins are highly conserved across Vertebrate phylogeny.

### *TOPAZ1* is specifically expressed in gonads

**Figure 4:**
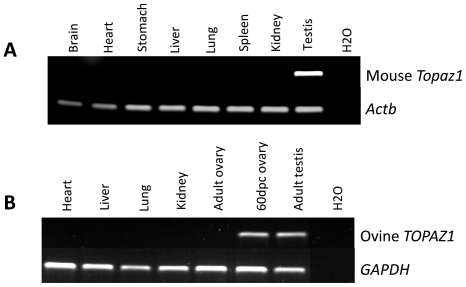
Expression of *TOPAZ1* mRNA in different sheep (A) and mouse (B) tissues. (**A**) The expression of sheep *TOPAZ1* mRNA was tested using RT-PCR in four adult somatic tissues (heart, liver, lung and kidney) and in gonads (60 d*pc* ovary and adult testis). (**B**) The expression of the mouse *Topaz1* gene was tested in seven adult somatic tissues (brain, heart, stomach, liver, lung spleen and kidney) and in adult testis. *GAPDH* (glyceraldehyde-3P-dehydrogenase) and *Actb* amplification served as loading controls.

The tissue specificity of both sheep and mouse *TOPAZ1/Topaz1* transcripts was tested by semi-quantitative RT-PCR analyses on different adult somatic tissues and gonads (adult and fetal ovaries in sheep). The primers used are listed in **Table S4**. *GAPDH* in sheep and *βeta-actin (Actb)* in mouse served as positive controls and were detected in all tissues (**Figure 4**). Among the different tissues tested, mouse *Topaz1* cDNA was exclusively expressed in adult testis (**Figure 4A**) and sheep *TOPAZ1* cDNA was detected in both adult testis and fetal ovary (**Figure 4B**). None of the somatic tissues tested displayed amplified bands corresponding to *TOPAZ1* cDNA in both mouse and sheep species.

### *TOPAZ1* displays a time-regulated expression profile during gonad development

**Figure 5:**
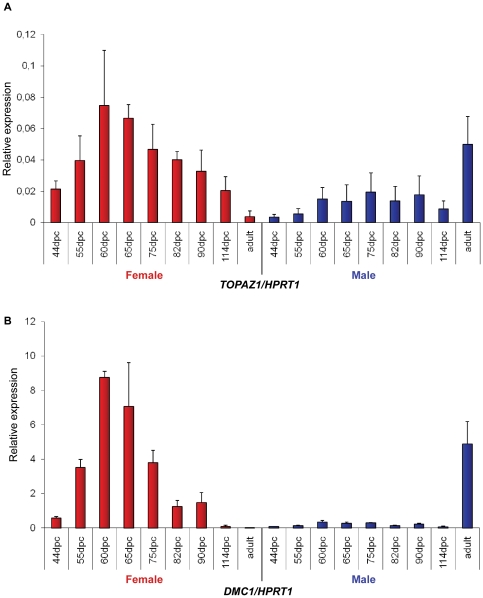
Expression of *TOPAZ1* and *DMC1* mRNA in sheep gonads at different developmental stages. Quantification of *TOPAZ1* (**A**) and *DMC1* (**B**) genes expression using quantitative real-time RT-PCR analysis at several fetal stages (44, 55, 60, 65, 75, 82, 90, 114 d*pc*) and in adult ovary (red histograms) and testis (blue histograms). The *HPRT1* (hypoxanthine phosphoribosyltransferase) gene was used as a reporter gene. Values indicated on the graph are means ± SEM of two independent RT experiments at each stage.

In order to study the time course of *TOPAZ1* mRNA expression, quantitative RT-PCR was performed (primers listed in **Table S5**) on female and male gonad tissues at different stages of development (fetal, post-natal and adult). The relative expression of sheep female *TOPAZ1* mRNA, normalized to *HPRT1* mRNA expression (**Figure 5A**), increased during the meiosis prophase I period (55–65 d*pc*), decreased until the end of the fetal life and then remained very low in adult ovary. Throughout the fetal period of testicular development, the relative expression of *TOPAZ1* mRNA was low. By contrast, *TOPAZ1* expression was only high in the adult testis of sheep.

According to the expression profile of ovine *TOPAZ1* (**Figure 5A**) i.e. its increase during the 55–75 d*pc* period in fetal ovary and in adult testis, we compared its expression with that of *DMC1*, a gene essential for meiotic homologous recombination during the development of male and female gonads in sheep using quantitative RT-PCR (**Figure 5B**). In females, *DMC1* transcripts were detected as from 55 d*pc*, peaked between 60–65 d*pc* and then decreased after 75 d*pc*. This profile was very similar to that of *TOPAZ1*. In males, the expression of both *DMC1* and *TOPAZ1* was strongly observed at the adult stage.

**Figure 6:**
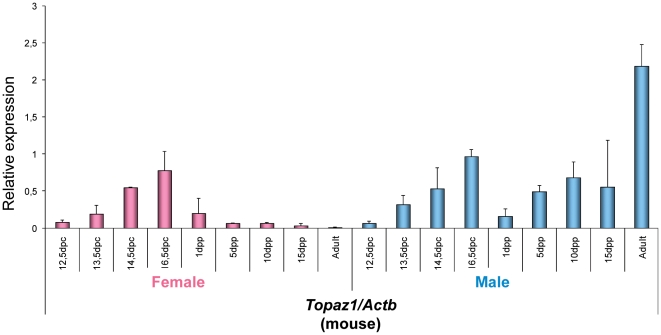
Expression of *Topaz1* mRNA in mouse gonads at different developmental stages. Quantification of *Topaz1* gene expression using quantitative real-time RT-PCR analysis at several fetal stages (12.5, 13.5, 14.5, 16.5 d*pc*), after birth (1, 5, 10, 15 d*pp*) and in adult ovary (pink histograms) and testis (light blue histograms). The *Actb* gene was used as a reporter gene. Values indicated on the graph are means ± SEM of three independent RT experiments at each stage.

In the mouse (**Figure 6**), the relative expression profile of *Topaz1* mRNA differed from that seen in sheep. Indeed, in developing female and male gonads in fetal mice, the relative expression of *Topaz1* mRNA was approximately the same in both sexes, and further increased during development. In females, its relative expression was decreased after birth; it was even quite low at 5 days *post-partum* (d*pp*) and almost null in mouse adult ovaries. In males, *Topaz1* mRNA increased as from 5 d*pp* to become strongly expressed in the adult mouse testis.

Thus *TOPAZ1/Topaz1* was a gonad-specific expressed gene in the sheep and mouse. However, its expression profile differed slightly between these two species. Contrary to the mouse, the sheep *TOPAZ1* transcript was preferentially expressed in females during fetal life when prophase I meiosis occurred (**Figures 5A and 5B**). Indeed, we had previously shown that the leptotene and zygotene stages were clearly identifiable in sheep ovogonia as from 55 d*pc* [51]. At this stage, the transcription of *DMC1*, *SPO11*, and *MSH4* was initiated. A strong signal was observed until 75 d*pc*, after which expression decreased more or less rapidly and was found to be present at a low level at 94 d*pc*. At birth, all ovocytes were at the dictyate stage of meiosis [51]. The sheep *TOPAZ1* transcript presented a profile similar to that of meiotic genes.

### *Topaz1* is a germ cell-specific gene

**Figure 7:**
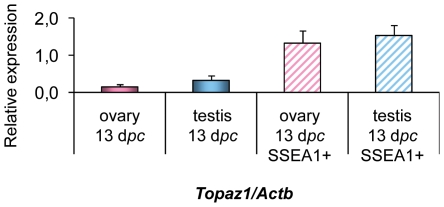
Expression of the *Topaz1* transcript in mouse enriched germ cell fractions. Germ cells were isolated from 13 d*pc* gonads by means of SSEA1+ immunomagnetic isolation. Quantification of *Topaz1* gene expression using quantitative real-time RT-PCR analysis in whole ovaries (pink histogram) and testes (light blue histogram) and in germ cell fractions (hatched histograms). Values indicated on the graph are means ± SEM of three independent samples.

In order to determine whether *Topaz1* expression in gonads is restricted to germ cells, we analyzed germ cell fractions from mouse testis and ovary. Enrichment in germ cells was ensured by the immunomagnetic isolation of SSEA1+ cells (SSEA1-enriched germ cells) from fetal gonads at 13 d*pc*. Stage-Specific Embryonic Antigen 1 (SSEA1) is regulated developmentally during early embryogenesis and is widely used as a marker to monitor the differentiation of pluripotent EGCs, ESCs and embryonic carcinoma cells (ECCs) [58]. To quantify *Topaz1* gene expression, we performed RT-qPCR on whole gonads versus SSEA1-enriched germ cells from male and female 13 d*pc* gonads (**Figure 7**). *Topaz1* transcripts were abundant in both male and female SSEA1-enriched cells but were rare in whole gonads, showing that the *Topaz1* gene was germ cell-specific in the mouse.

**Figure 8:**
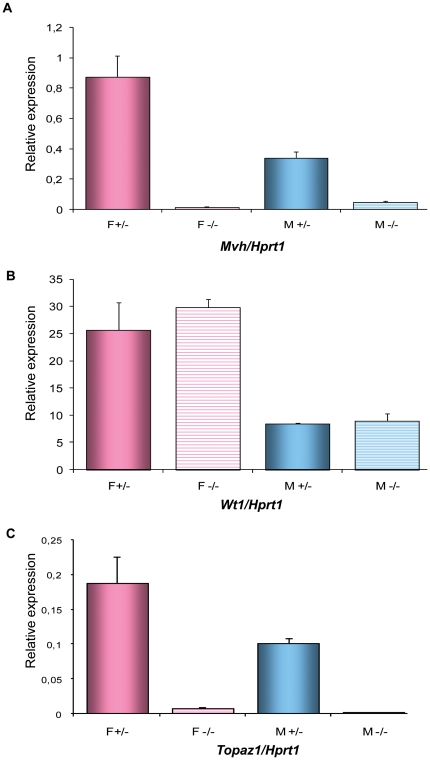
Expression of *Topaz1, Mvh* and *Wt1* transcripts in mouse WlacZ/+ or WlacZ/WlacZ gonads. (A) Expression of *Mvh,* a gene only expressed in germ cells, was used to verify the absence of germ cells from WlacZ/WlacZ gonads (F−/− and M−/−, hatched histograms) in contrast to WlacZ/+ gonads (F+/− and M+/−, plain histograms). (B) Expression of the *Wt1* gene in somatic cells was used to verify the integrity of somatic cells. (C) The expression of *Topaz1* was determined by qRT-PCR analysis in WlacZ/+ or WlacZ/WlacZ ovary and testis. The *Hprt1* gene was used as a reporter gene. Values indicated on the graph are means ± SEM of two independent samples.

To consolidate our findings, we used homozygous fetuses with null mutation for the *c-Kit* gene [59]. The absence of *Kit* prevents the migration and survival of PGC, so that gonads from these fetuses contain no germ cells. To produce these WlacZ/WlacZ embryos, we intercrossed WlacZ/+ mice (obtained from Transgenesis Institute of Orléans-Villejuif Resource Center, (http://transgenose.cnrs-orleans.fr/eng/intragene/presentation.php). An analysis of *Topaz1* mRNA expression within a pool of gonads from WlacZ/WlacZ embryos (from 17.5 to 19.5 d*pc*) was compared with the expression of *Vasa* (or *Mhv*, germ cell marker) and *Wt1* (Wilms tumor 1, somatic cell marker) (**Figure 8**). *Topaz1* transcripts (such as *Vasa*) were clearly absent from male and female germ cell-deprived gonads. On the other hand, *Wt1*, a gene with somatic expression, was highly expressed in these gonads. These findings reinforced our previous data indicating that *Topaz1* is a germ cell-specific gene in the mouse.

### *Topaz1* expression is not RA-dependant in mouse fetal testis

**Figure 9:**
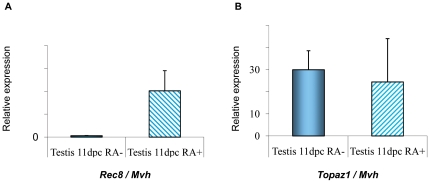
Effect of retinoic acid (RA) on *Topaz1* expression in mouse fetal testis. Mouse testes at 11 d*pc* were cultured for 3 days with (RA+, hatched histograms) or without (RA−, plain histograms) retinoic acid (10^−6^ M). At the end of the culture period, total RNA was extracted and *Rec8* (**A**) and *Topaz1* (**B**) mRNA expressions were measured by real-time quantitative RT-PCR. Values indicated on the graph are means ± SEM of three independent samples.

In order to determine whether *TOPAZ1* could be regulated by the retinoic acid (RA) pathway, *in vitro* mouse fetal testis culture experiments were performed [18]. Indeed, whereas *in vivo* male meiosis only starts after birth, RA has been shown to induce meiosis *in vitro* in mouse fetal germ cells from both 11 and 12.5 d*pc* XY gonads [18]. In these *in vitro* cultures, about 20% of the germ cells displayed histological characteristics typical of the leptotene and zygotene stages. After such *in vitro* culture experiments on 11 d*pc* mouse testis, the expression of *Rec8* (a meiosis-specific phosphoprotein involved in recombination events), and of *Topaz1,* was quantified using real-time quantitative RT-PCR (**Figure 9**). We showed that a high level of RA (10^−6^ M) induced the onset of meiosis (as attested by the increase in *Rec8* expression) after three days of culture, when compared with the control culture (without RA). By contrast, the expression of *Topaz1* was not modified by RA treatment and *Topaz1* expression in the mouse fetal testis did not require RA signaling.

### Production of a specific TOPAZ1 antibody

**Figure 10:**
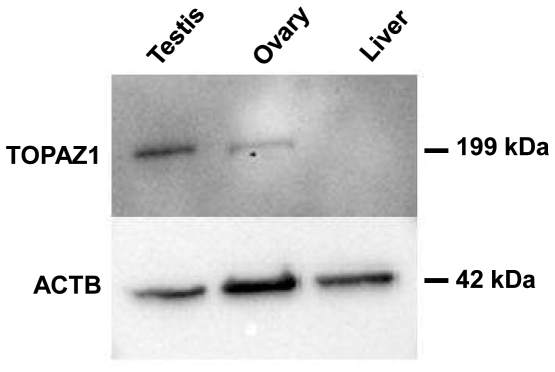
Specificity of anti-TOPAZ1 antibody by Western blot. Western-blotting experiments were performed with cytosolic extracts from sheep adult testis, fetal ovary and liver. Anti-TOPAZ1 antibody recognized a 199 kDa protein in sheep adult testis and 60 d*pc* ovary, but no band was detected in sheep liver. As a positive control, ACTB expression was detected in each sample.

To determine the cell type and sub-cellular localization of TOPAZ1 protein, we produced a polyclonal antibody from two 15-mer peptides. The specificity of this antibody was tested by Western blot analysis on protein extracts from different sheep tissues (cytosolic fractions of adult testis, fetal ovary at 60 d*pc* and liver). This analysis showed that TOPAZ1 antibody bound specifically to adult testis and fetal ovary extracts, but not to liver extracts (**Figure 10**). The molecular weight of TOPAZ1 protein was approximately 199 kDa, slightly higher than the predicted molecular weight of 180 kDa. This difference may have resulted from post-translational modifications. No similar signals were detected in nuclear extracts from sheep gonads (data no shown). The antibody against BETA-ACTIN (ACTB) detected a specific 42 kDa band in all the extracts tested, thus serving as a positive control. TOPAZ1 protein was exclusively expressed in the cytosolic compartment of gonad cells, and the protein level was higher in sheep adult testis than in the fetal ovary. These results were consistent with our RT-qPCR analysis (**Figure 5A**).

### TOPAZ1 protein is present in germ cells of sheep fetal ovary

**Figure 11:**
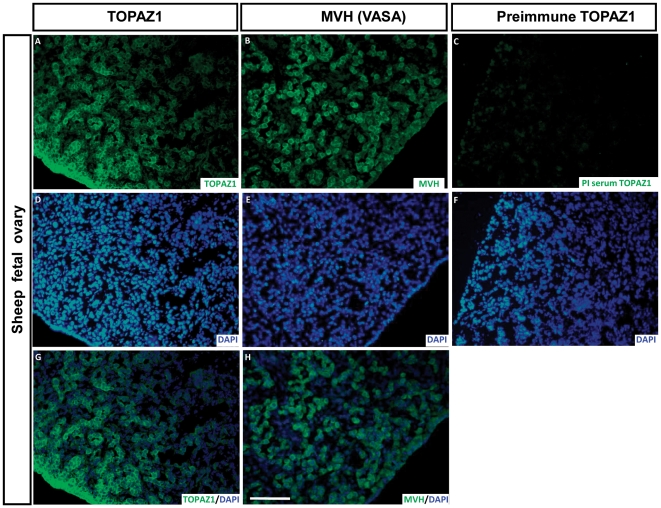
Immunodetection of TOPAZ1 protein in sheep fetal ovary. Immunofluorescence was performed on transversal sections of sheep 60 d*pc* ovaries to detect TOPAZ1 (A) and MVH (B) proteins. Pre-immune serum (PI) was also tested (C). For each section, DAPI was performed to detect nuclei (D-E-F). TOPAZ1 showed similar cellular localization as MVH (A, B). The immunolabeling of TOPAZ1 and MVH was merged with DAPI (G, H). TOPAZ1 can be detected in the cytoplasm of germ cells in ovine fetal ovary. PI serum of TOPAZ1 did not reveal any staining (C). Scale bars = 200 µm in H (applies to A–G).

The TOPAZ1 antibody was used on sheep fetal ovary sections at 60 d*pc* (**Figure 11A, G**), the stage displaying the peak expression of *TOPAZ1* mRNA (**Figure 5A**). We also performed immunodetection of MVH (VASA) (a germ cell marker expressed in the cytoplasm of germ cells) on adjacent sections of 60 d*pc* ovary (**Figure 11B, H**). Immunofluorescence was mainly detected in the germ cells. When merged with DAPI, TOPAZ1 staining was clearly localized in the cytoplasm of the cells (**Figure 11G**). When comparing TOPAZ1 and MVH immunostaining, we concluded that TOPAZ1 was expressed in the cytoplasm of 60 d*pc* female germ cells in sheep. Pre-immun TOPAZ1 was also tested on sheep 60 d*pc* ovary sections and did not reveal any staining (**Figure 11C**).

### TOPAZ1 protein is present in germ cells of mouse adult testis

**Figure 12:**
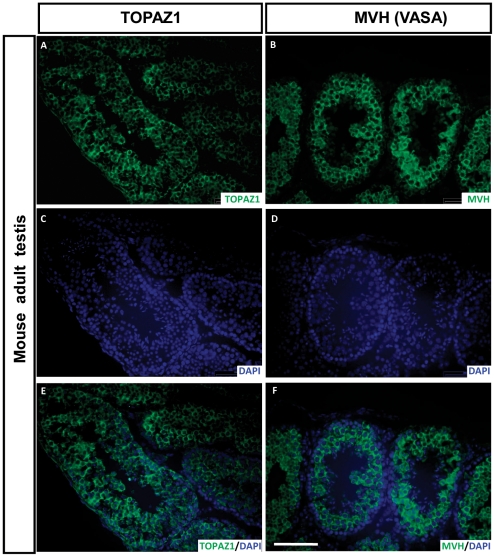
Immunodetection of TOPAZ1 protein in mouse adult testis. Immunofluorescence was performed on sections of mouse adult testis to detect TOPAZ1 (A) and MVH (B) proteins. Anti-TOPAZ1 antibody raised against sheep protein was used to detect mouse Topaz1. For each section, DAPI was performed to detect nuclei (C–D). Mouse TOPAZ1 displayed a localization similar to that of MVH (A, B). The immunostaining of TOPAZ1 and MVH was merged with DAPI (E, F). TOPAZ1 can be detected in the cytoplasm of germ cell in mouse testis. Scale bars = 200 µm in H (applies to A–F).

The localization of TOPAZ1 in mouse adult testis was determined using immunofluorescence (**Figure 12A, E**). The peptides used for immunization were designed according to the sheep sequence and were not fully homologous with the mouse *Topaz1* sequence. In order to enhance antibody specificity, purified anti-TOPAZ1 antibody was used on mouse sections. TOPAZ1 displayed clear staining in germ cells of from mouse adult testis (**Figure 12A**). The same degree of staining was achieved using MVH antibody (**Figure 12B** **)**. Moreover, when merged with DAPI, the TOPAZ1 protein appeared to be clearly localized in the cytoplasm of these germ cells (**Figure 12G**). Our results thus confirmed the cytoplasm localization of TOPAZ1 in the germ cells of mouse adult testis, a funding in agreement with those obtained by the expression of *Topaz1* transcripts in the germ cell enriched fraction (**Figure 7**) and in germ cell-deprived gonads (**Figure 8**). *TOPAZ1*/*Topaz1* mRNA and TOPAZ1 protein were localized in the germinal cell lineage of sheep and mice of both sexes.

## Discussion

During this study, we isolated and characterized a new gene encoding for a putative protein containing a PAZ domain. Until now, the evolutionarily conserved PAZ (Piwi/Argonaute/Zwille) domain had been found in two families of proteins that are involved in post-transcriptional gene silencing [60], the PIWI and DICER families. The three-dimensional structure of this domain has been solved [61]–[63] and studies have revealed a unique mode of single-stranded nucleic acid binding in which the two 3-terminal nucleotides are buried in a hydrophobic cleft. It has been proposed that the PAZ domain contributes to the specific recognition of siRNAs [64].

In addition, TOPAZ1 contained a zinc-binding domain of the Cys-X_9_-Cys-X_4_-Cys-X_3_-His type (termed the CCCH domain). Berg *et al.* defined that the CCCH family is a group of zinc-finger proteins consisting of a canonical C-X_6–14_-C-X_4–5_-C-X_3_-H motif [65] http://www.ncbi.nlm.nih.gov/pubmed/8599083. After analyzing the entire CCCH family in Arabidopsis and rice, Wang *et al.* re-determined that CCCH proteins are characterized by one to six C-X_4–15_-C-X_4–6_-C-X_3_-H motifs which constitute glycine-rich and phenylalanine-rich sequences [66]. CCCH-type zinc-finger proteins are RNA binding proteins with regulatory functions at all stages of mRNA metabolism, including pre-mRNA splicing, mRNA transportation, sub-cellular localization and stability/degradation, transcription, ubiquitination, and poly ADP-ribosylation [67], [68]. It has been shown that different CCCH-type ZnF proteins interact with the 3′-untranslated region of various mRNA [69], and that a single CCCH domain is capable of binding single-stranded RNA with considerable affinity and selectivity [70]. The presence of these two domains in the TOPAZ1 protein thus strongly suggested a role for this factor in RNA recognition and binding.

Phylogenetic analysis has revealed a fish-specific TOPAZ1 clade within vertebrates. Among mammals, eutherians are separated into two groups, rodents and others. This divergence could explain the difference in expression patterns that we observed between mice and sheep during gonad development. The strong conservation of the ZnF_CCCH domain in fish and amphibians suggests that the TOPAZ1 molecules of lower vertebrates possess the structural features required for RNA binding. Whilst the C-terminal from the ZnF_CCCH domain is well conserved among all Topaz1 molecules, the N-terminal before the ZnF_CCCH domain is more divergent between fish, amphibians, monotremes and eutherian mammals (data not shown).

During the current study, we showed that the *TOPAZ1/Topaz1* gene was specifically expressed in the gonads of sheep and mice, in a sex-specific manner. Its expression was stronger in females than in males during fetal gonad development. However, this pattern was reversed after birth, with strong expression in adult males only and the disappearance of Topaz1 expression from the adult ovary. This time course was suggestive of a relationship between Topaz1 expression and the occurrence of germ cell meiosis. To reinforce this hypothesis, we analyzed the expression profile of *DMC1* in the same samples and compared its expression pattern in sheep. *DMC1* is a meiosis-specific *RecA/Rad51* homolog required for the recombinational repair of meiotic Double Strand Breaks (DSBs). The DMC1 protein was detected in mouse leptotene-to-zygotene spermatocytes at the initiation of homolog pairing [71]. In *Saccharomyces cerevesiae* and mice of both sexes, *DMC1*-deficient mutants arrest in the late zygoneme/early pachyneme of meiotic prophase I, with an accumulation of DSBs and defective formation of the synaptonemal complex [72], [73]. The comparison of sheep *TOPAZ1* and *DMC1* expression patterns revealed a very similar profile. Furthermore, we demonstrated that *Topaz1* was germ line-specific using two different approaches; the enrichment of mouse germ cells by SSEA1 antibody and the use of gonads from WlacZ/WlacZ mutant mice devoid of germ cells. Moreover, we showed that TOPAZ1/*Topaz1* mRNA encoded a protein present in germ cells from both male and female gonads. Taken together, these results reinforce the idea that *TOPAZ1/Topaz1* could be involved in germ cell development.

Our study also showed that *Topaz1* expression was not stimulated by retinoic acid in cultured male fetal gonads from mice. This could have been due either to the incomplete meiosis prophase I obtained during our organ culture experiments [18] or to the need for non-retinoid pathways in order to regulate *Topaz1* expression [28].

In addition to the recombination events and chromatin exchange that occur during meiosis prophase I, it is of paramount importance for germ cell genomes to be protected from the uncontrolled propagation of mobile genetic elements. These mobile elements must be distinguished from endogenous genes and then selectively silenced. In *Drosophila*, germ cells express a class of small RNAs (piRNAs) that are specialized in the repression of mobile elements [74]–[76]. Germ cells in mice express two types of piRNAs: (*i*) a small fraction of piRNAs derived from the repetitive genomic regions expressed during mouse embryogenesis, and (*ii*) a larger fraction of piRNAs derived from non-repetitive regions that start to be expressed during the pachytene stage of meiosis (pachytene piRNAs). [46], [47], [77]. Pachytene piRNAs interact with MIWI (mouse PIWI) and MILI (MIWI-like), two of the three mouse PIWI proteins [47]. In mice, the PIWI clade contains three members: *Miwi2*, *Mili*, and *Miwi*, all of which display distinctive developmental expression patterns [78]–[81]. In the male germline, a deficiency in one of two Piwi family members, *Mili* or *Miwi2*, results in the loss of DNA methylation marks on transposons, and mutant animals display a phenotype similar to that of *Dnmt3L*-deficient mice [81]–[84]. These data have led to the hypothesis that Piwi/piRNA complexes might serve as sequence-specific guides that direct the *de novo* DNA methylation machinery to transposable elements [81], [82], [85]. The fact that *Topaz1* possesses two RNA interaction domains and an expression profile related to meiosis prophase I supports the hypothesis that *Topaz1* might be involved in the piRNA pathway. It is noteworthy that *MOV10L1*, a transcript previously isolated in our sheep fetal ovary libraries [50] has recently been confirmed as an essential component in the Piwi-interacting RNA pathway in the mouse [53], [54]. On the other hand and by comparison with MVH immunostaining, we showed that TOPAZ1 displayed a similar cellular localization. Consequently, *TOPAZ1* could be expressed in primordial germ cells and in germ cells undergoing gametogenic processes until the post-meiotic stages in both males and females. In adult testis, MVH and TOPAZ1 proteins were located in the cytoplasm of spermatogenic cells. It has recently been shown that MVH plays a crucial role in piRNA processing and in the gene silencing of retrotransposons [86]. The similarity of the expression profiles and cell localization of both genes, in addition to the presence of PAZ and RNA-binding domains in Topaz protein, reinforce the possible role of TOPAZ1 in the regulation of retrotransposons in the germ line.

Further studies are necessary to investigate the functional role of TOPAZ1 in mammalian gametogenesis, and for this purpose we are currently generating a *Topaz1* knockout mouse.

In conclusion, *TOPAZ1* is a new evolutionarily conserved gene, specifically expressed in gonads and probably involved in gametogenesis. The presence of a PAZ domain in Topaz1 suggests that it could be involved in the RNA silencing pathway. It constitutes a potential new actor in piRNA-directed retrotransposon silencing in germ cells that contributes to a safeguard mechanism for genetic information.

## Materials and Methods

### Animal and tissue samples

Pregnant Pré-Alpes female sheep were obtained as previously described [50], [51]. Sheep fetuses were collected at 44, 55, 60, 65, 75, 82, 90 and 114 days *post coitum* (d*pc*). The gonads of adult animals were collected at slaughter, cut into small pieces, flash frozen in liquid nitrogen and then stored at −80°C. Other organs from sheep fetuses, such as the liver, kidney, lung and heart, were treated similarly. C57/Bl6, 129/Sv and NMRI mouse fetuses and adult organs were also collected, frozen immediately and then stored at −80°C. Experiments were performed in accordance with the International Guiding Principles for Biomedical Research involving animals, as promulgated by the Society for the Study of Reproduction and with the European Convention on Animal Experimentation. All researchers working directly with the animals possessed an animal experimentation license delivered by the French veterinary services, and the permit number for our studies is C91332101. The study also involved the use of 129/Sv mice carrying the KitW/LacZ allele. The *KIT* gene was inactivated and the first exon of *Kit* was replaced by an nlsLacZ-neo cassette. Full details on this construction can be found in the paper by Bernex and colleagues [59]. Ovaries and testes from heterozygous (WlacZ/+) and homozygous (WlacZ/WlacZ) mice were collected at between 17.5 d*pc* and 20.5 d*pc*.

### RNA extraction, reverse transcription (RT), semi-quantitative RT-PCR and real-time PCR

Total sheep RNAs were extracted from each sample using Trizol® reagent (Invitrogen Life Technologies, Cergy-Pontoise, France] plus the RNeasy Mini kit (QIAGEN SA, Courtaboeuf, France), following the manufacturer's instructions. In sheep, we used six ovaries and four testes at 45 d*pc*, four gonads at 55 d*pc*, two gonads at 60-65-75-82 d*pc*, one gonad at 90–114 d*pc* and a sample of adult gonads. RT was performed on each sample using 5 µg Dnase-treated RNA incubated with random hexanucleotide primers with Superscript II (Invitrogen, Cergy-Pontoise, France), according to the manufacturer's instructions.

Briefly, semi-quantitative RT-PCR was performed on 1 µL (corresponding to 25 ng reverse-transcribed total RNA) of each RT, which was amplified using 0.5 U Taq polymerase (TaKaRa, Lonza, Verviers, Belgium). The primer sequences and PCR conditions used are shown in **Table S4**. In sheep, the *GAPDH* gene was used as a control. The PCR conditions were presented in **Table S4**.

Real-time PCR analysis of *TOPAZ1* was performed using the ABI Prism 7700 HT apparatus (Applied Biosystems). Briefly, PCR was performed with the ABsolute blue QPCR SYBR Green ROX mix (Abgene, Les Ulis, France), using 50 ng of cDNA from the RT. The primers used for real-time PCR are presented in **Table S5**. We have performed all control experiments to ensure that our primers could not amplify any genomic products. All expression data were normalized using the *HPRT1* expression level for sheep samples. Real-time PCR on sheep involved two different RT experiments for each stage and the SEM was calculated from these two independent samples. Real-time PCR in the mouse was performed on three different RT samples for each stage.

In the mouse, total RNA was extracted using the RNeasy Mini kit (Qiagen) in C57/Bl6 and NMRI mice or the RNeasy Micro kit (Qiagen) in 129/Sv WlacZ/+ and WlacZ/WlacZ mice, depending on the size of the gonads. Ovaries from WlacZ/+ or WlacZ/WlacZ mice at between 17.5 d*pc* and 20.5 d*pc* were pooled in two separate tubes (14 and 18 ovaries, respectively). The same procedure was used for WlacZ/+ and WlacZ/WlacZ testes (4 and 6 testes for each condition). Reverse transcription was carried out with the Omniscript kit (Invitrogen), according to the manufacturer's instructions. The expression of *Topaz1* or *Rec8* genes was assessed by real-time PCR using the SYBR Green Universal PCR Master Mix 2× (Applied Biosystems, Foster City, CA). Reactions were carried out and fluorescence was detected on an ABI Prism 7000 apparatus. In the mouse, the amount of each cDNA detected was normalized using *Actb, Hprt1 or Mvh*. All primers are presented in **Table S5**.

### 5′RACE-PCR in sheep

The full-length cDNA of sheep *TOPAZ1* was determined using the 5′ RACE System (Invitrogen, Cergy-Pontoise, France). Total RNA was extracted from 60 d*pc* female fetal gonads. One microgram of total RNA was reverse-transcribed into first-strand cDNA using a specific oligo for *TOPAZ1* (TOPAZ1_RT, **Table S1**) and Superscript II (Invitrogen, Cergy-Pontoise, France). First-strand cDNA was treated with RNase H (Invitrogen, Cergy-Pontoise, France) and purified on a S.N.A.P column (Invitrogen, Cergy-Pontoise, France). TdT tailing was performed on cDNA using dCTP and terminal transferase (Invitrogen, Cergy-Pontoise, France). Thirty two PCR cycles were performed using the AAP anchor primer (a non-specific primer) and *TOPAZ1* specific primer 1, with or without 5% formamide. A second PCR were performed on 5 µL of the PCRI samples diluted at 1/100, with two different *TOPAZ1*-specific primers (Topaz1–2 or Topaz1–3 nested probe, **Table S1**) and AUAP (a non-specific primer). Both PCR procedures were performed according to the following protocol: 94°C for 30 s, 55°C for 30 s, and 72°C for 1.5 min for 32 cycles, followed by an extension for 10 min at 72°C. All RACE-derived PCR products were subcloned into the pGEMTeasy vector and the clones were then sequenced.

Primers for *TOPAZ1* cDNA in sheep and mouse were designed to verify the size of the full-length cDNA sequences on adult testis. They are listed in **Tables S2 and S3**. The PCR conditions were 94°C for 30 s, 60°C for 30 s, and 72°C for 1.5 min for 35 cycles, followed by an extension for 10 min at 72°C.

### NMRI Mouse Testis cultures with retinoic acid (RA)

Pregnant female mice were killed by cervical dislocation at 11 d*pc* and their fetuses removed from the uterine horns. Gonads and their mesonephros were isolated from the fetuses under a binocular microscope and kept in Ham F12/DMEM (1∶1) (Life Technologies, Inc., Grand Island, NY, USA) until explantation. The sex of the fetus was determined by PCR amplification of *Sry*, as previously described [87]. The gonads were cultured on Millicell-CM Biopore filters (Millipore, Billerica, MA, USA) in 0.3 ml of Ham F12/DMEM (1∶1) for 3 days under a 5% CO_2_/95% air atmosphere. The medium was changed every 48 h. One gonad from each fetus was cultured in a medium containing all-trans-RA (10^−6^ M, Sigma, St Louis, MO), and the other was cultured in a control medium.

### Purification of NMRI mouse germ cells

About 50 gonads from 13.5 d*pc* fetuses were digested first in 0.25% trypsin-0.02% EDTA (Trypsin/EDTA solution, Sigma-Aldrich, St. Louis, MO) at 37°C. The samples were then centrifuged and digested with 2 mg/ml collagenase and 0.02 mg/ml DNase I in HBSS. For the purification of SSEA1-positive cells, dispersed cells were incubated with anti-SSEA1 (1/5, anti-SSEA1 monoclonal antibody, DSHB, Iowa) in PBS, 0.5% BSA (PB) for 20 min at 4°C. The cells were then centrifuged and washed once with 1 ml PB, before being incubated with 20 µL microbead-linked donkey anti-mouse IgM antibody (Miltenyi Biotec, Germany) in 300 µL PB with 2 mM EDTA (PBE) for 15 min at RT. The column was rinsed three times with 500 µL PBE to wash out unbound cells, which represented the SSEA1- cell fraction. After removal from the magnet, the column was flushed with 1 ml PB, which allowed collection of the SSEA1+ cell fraction. The cells were pelleted, RLT buffer was added and total RNA was extracted as previously described.

### Phylogenetic analysis

*TOPAZ1* sequences were retrieved by BLAST analysis from the databases accessible via the NCBI (http://blast.ncbi.nlm.nih.gov/Blast.cgi) and ENSEMBL (http://www.ensembl.org/Multi/blastview) servers. Sequence analysis was performed using the GCG Wisconsin package (Version 11.1, Accelrys Inc., San Diego, CA). Gene structure and protein sequences were predicted from genomic sequences using FGENESH [88]; (http://linux1.softberry.com/berry.phtml?topic=fgenesh&group=programs&subgroup=gfind). Multiple sequence alignments were generated using ClustalX [89]. Phylogenetic analysis was performed on a 620 amino-acid alignment extending from the CCCH zinc finger to the C-terminal end using the neighbor-joining method [57]; (1,000 bootstrap replicates; Kimura two-parameter correction) as implemented under Seaview (Version 4.0) [90].

### Proteins Extraction

Frozen tissues (sheep 60 d*pc* liver and ovaries and adult testis) were pounded into liquid nitrogen. The tissue powder was then mixed with 1 ml lysis buffer in a Dounce, and 0.2% NP40 was added. Samples were centrifuged at 13,000 rpm for 1 hr at 4°C, and the supernatant corresponding to the cytosolic extract was removed. The nucleus pellet was resuspended in 100 ml nuclear protein extraction buffer (20 mM Hepes pH 7.7, 1.5 mM MgCl2, 0.2 mM ethylenediaminetetraacetic acid (EDTA), 25% glycerol, 1 mM NaF, 1 mM Na3VO4, 10 mM ammonium molybdate, 0.5 mM dithiothreitol (DTT), 2 mM benzamidine, 0.5 mM phenylmethyl sulfonyl fluoride (PMSF)) to which 10 ml of 4 M NaCl was added. After 1 hr of gentle mixing at 4°C, the samples were centrifuged for 30 min at 13,000 rpm, and the supernatant corresponding to nuclear extracts was collected. The Bradford method was used for protein quantification and samples were conserved at −80°C.

### Generation of an anti-TOPAZ1 antibody

Anti-TOPAZ1 antibody was generated by Eurogentec Company. Two peptides of 15 amino acids were used to immunize a rabbit: TEKRKEINAKSSERE (sheep amino acids 601–615) and SLSGAEVRQSRTKEN (sheep amino acids 625–639), chosen for their potential high antigenicity. Their homologies with mouse Topaz1 counterparts were 60% and 66%, respectively. The antibody was affinity purified with both peptides by Eurogentec Company. The non-purified antibody was used in sheep and the purified antibody was used in mouse.

### Western Blotting

For Western-blot analyses, (11 mg) proteins were separated in NuPAGE 4–12% Bis-Tris polyacrylamide gels according to the manufacturer's instructions (Invitrogen) and transferred onto a PVDF membrane (Millipore). The blots were incubated with primary antibodies (rabbit polyclonal anti-TOPAZ1 antibody 1∶300, or rabbit monoclonal anti-ACTB, 1∶500, Sigma) overnight at 4°C and were then incubated for 1 h with the corresponding peroxidase-conjugated antibody (anti-rabbit immunoglobulin, 1∶5000, Biosystem). Immunoreactive bands were detected by ImmobilonTM western Chemiluminescent HRPSubstrate (Millipore).

### Immunofluorescence

Freshly dissected gonads were fixed in 4% paraformaldehyde in phosphate saline buffer (PBS) at 4°C overnight. After washing in PBS with increasing concentrations of sucrose (0, 12%, 15%, and 18%), the tissue specimens were embedded in Jung Tissue Freezing Medium (Leica Instruments) and frozen at 80°C. Cryosections (5 µm) were obtained and stored at −80°C. The sections were air-dried, treated with chloroform for 30 sec, and then rehydrated in PBS for 10 min. The TOPAZ1 antibody (1∶1000) and VASA antibody (or MVH, 1∶500, ab13840, Abcam) were diluted in 1% BSA/PBS and applied to tissue sections overnight at 4°C. After several washes, the sections were incubated with a secondary anti-rabbit IgG-FITC antibody against rabbit (1∶200, Vector) for 1 h at room temperature. The slides were then rinsed in PBS, mounted in Vectashield mounting medium with DAPI (Vector) and observed with a Leica DMRB epifluorescence microscope coupled to a DP50CCD camera (Olympus).

## Supporting Information

Figure S1 **Main genes involved in follicle formation and preservation of germ cell resting pool.** Several factors are involved in primordial (FIGLA, NOTCH2, SOHLH1 and 2, FOXL2), primary (NOBOX, LHX8, cKIT/KL) or secondary (GDF9, BMP15) follicle formation. Others support the resting pool of primordial follicles and avoid their differentiation (AMH, PTEN, FOXO3A).
(PDF)

Figure S2 ***TOPAZ1*** **exon 1 in human, sheep and mouse.**
(PDF)

Table S1 **5′ RACE primer sequences used in sheep.**
(PDF)

Table S2 **Sequences of semi-quantitative RT-PCR primers used to sequence full-length** ***TOPAZ1*** **cDNA in sheep.**
(PDF)

Table S3 **Sequences of semi-quantitative RT-PCR primers used to sequence and/or verify the size of mouse** ***Topaz1*** **cDNA.**
(PDF)

Table S4 **Primer sequences and conditions used for RT-PCR analyses.**
(PDF)

Table S5 **Sequences of qPCR primers.** Oligonucleotides for qPCR were designed using the PrimerExpress Designer software (Perkin Elmer).
(PDF)
