# Supplementary figures and images for: TOPAZ1, a Novel Germ Cell-Specific Expressed Gene Conserved during Evolution across Vertebrates
Source: PLoS One. 2011 Nov 1;6(11):e26950. doi: 10.1371/journal.pone.0026950 (PMC3206057; doi:10.1371/journal.pone.0026950)

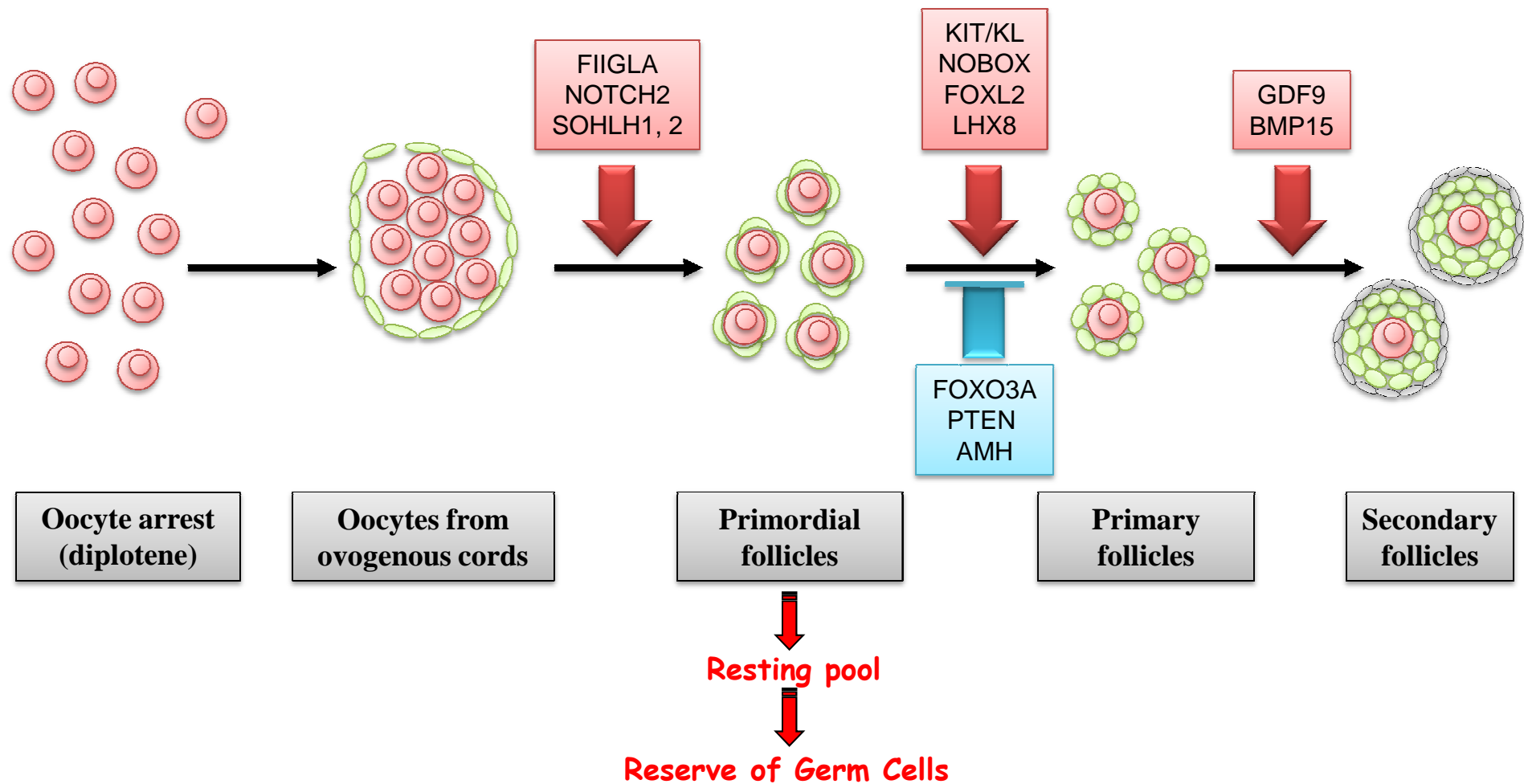

Supplement: Figure S1 — Main genes involved in follicle formation and preservation of germ cell resting pool. Several factors are involved in primordial (FIGLA, NOTCH2, SOHLH1 and 2, FOXL2), primary (NOBOX, LHX8, cKIT/KL) or secondary (GDF9, BMP15) follicle formation. Others support the resting pool of primordial follicles and avoid their differentiation (AMH, PTEN, FOXO3A). (PDF) [file pone.0026950.s001.pdf]

Figure S2: *TOPAZ1* exon 1 in human, sheep and mouse

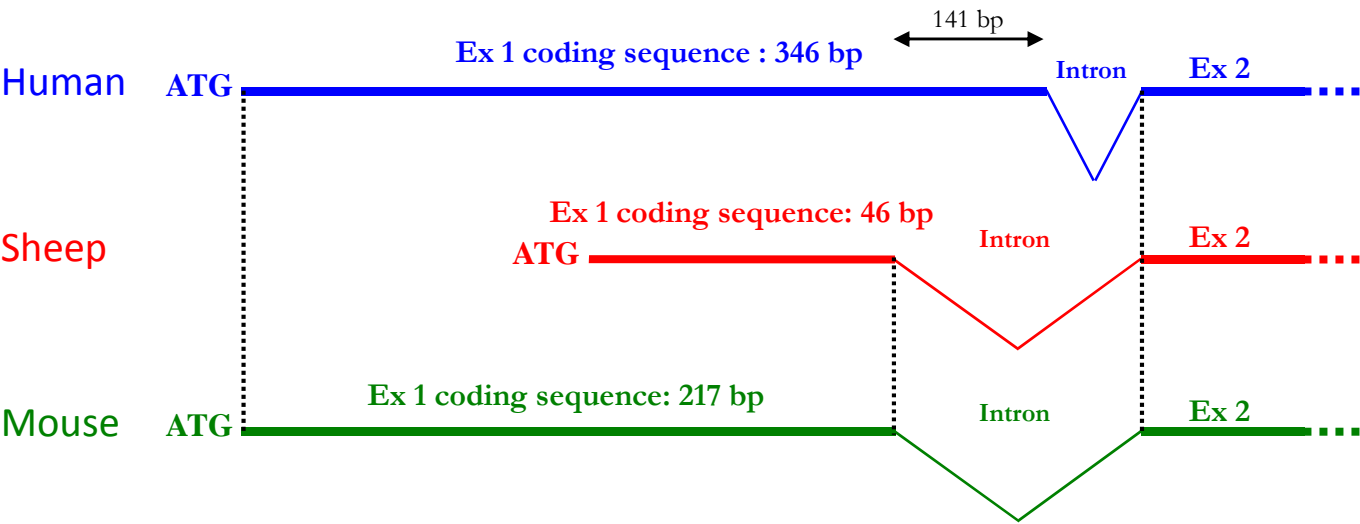

Supplement: Figure S2 — TOPAZ1 exon 1 in human, sheep and mouse. (PDF) [file pone.0026950.s002.pdf]
