# Supplementary material for: TOPAZ1, a Novel Germ Cell-Specific Expressed Gene Conserved during Evolution across Vertebrates
Source: PLoS One. 2011 Nov 1;6(11):e26950. doi: 10.1371/journal.pone.0026950 (PMC3206057; doi:10.1371/journal.pone.0026950)
Supplement: Table S1 — 5′ RACE primer sequences used in sheep. (PDF) [file pone.0026950.s003.pdf]

Table S1 - 5' RACE primer sequences used in sheep

**Type of Primers**	**Primers**
*TOPAZ1*_RT	5′-AGTCATTTTGAGAGCTGGGC-3′
TOPAZ1 specific primer 1	5′-CTACCAACCTTTCCTTACCC-3′
TOPAZ1-2	5′-TCACTGCATTCCACTGTTGG-3′
TOPAZ1-3	5′-CTGTGGATCATCACTTGAGG-3′
