# Supplementary material for: TOPAZ1, a Novel Germ Cell-Specific Expressed Gene Conserved during Evolution across Vertebrates
Source: PLoS One. 2011 Nov 1;6(11):e26950. doi: 10.1371/journal.pone.0026950 (PMC3206057; doi:10.1371/journal.pone.0026950)
Supplement: Table S2 — Sequences of semi-quantitative RT-PCR primers used to sequence full-length TOPAZ1 cDNA in sheep. (PDF) [file pone.0026950.s004.pdf]

Table S2 - Sequences of semi-quantitative RT-PCR primers used to sequence full-length *TOPAZ1* cDNA in sheep (ov, ovin)

Gene	Primers	Annealing temperature (°C)	Elongation time (sec)	Cycles	MgCl^2 (mM)	Size (bp)
ov*TOPAZ1* exon 1	5'-CCACGCGTCGACTAGTACGG-3'<br>5'-TTACCAGGTCAATCCCTGGC-3'	55	30	35	2,5	421
ov*TOPAZ1* exon 2A	5'-GAAGCCTCAAGTGATGATCC-3'<br>5'-GGTACATGCATACATCCTCC-3'	55	30	35	2,5	398
ov*TOPAZ1* exon 2B	5'-GGCTAC ACAAAACAGTAAGG-3'<br>5'-GTTCTTTTGCCAGTCATTGG-3'	55	30	35	2,5	912
ov*TOPAZ1* exon 2C	5'-TGAAAAGAGGTCTTCTAGGG-3'<br>5'-TCTCTCTCTGATGACTTGGC-3'	55	30	35	2,5	744
ov*TOPAZ1* exon 2D	5'-AGATACTGAACACAGGACGG-3'<br>5'-TGCTCTTCAGCAACATTGGG-3'	55	30	35	2,5	672
ov*TOPAZ1* exon 2-8	5'-CAGACCTCTTTGGAGTCTCC-3'<br>5'-AGTGGCTTCTGGAACACCCC-3'	59	30	35	2,5	652
ov*TOPAZ1* exon 8-15	5'-ACAGTGCAAGTTTGGTCACG-3'<br>5'-TCATTCCGATTCTTCCCAGC-3'	56	30	35	2,5	776
ov*TOPAZ1* exon 14-18	5'-GACTGTAAAGAAGAGCGACC-3'<br>5'-GCTCTGGCTTTGAGCCATAA-3'	56	30	35	2,5	677
ov*TOPAZ1* exon 18-20	5'-ACAGCCTTGGTATGTCATCC-3'<br>5'-ATCTTTCCACTGCAGCTTGG-3'	56	30	35	2,5	378
ov*TOPAZ1* exon 19-20	5'-GCTAGTAGTATTCAGAGTCC-3'<br>5'-AAAAGCCAAACCTTTCCGGC-3'	53	30	35	2,5	267
