# Supplementary material for: TOPAZ1, a Novel Germ Cell-Specific Expressed Gene Conserved during Evolution across Vertebrates
Source: PLoS One. 2011 Nov 1;6(11):e26950. doi: 10.1371/journal.pone.0026950 (PMC3206057; doi:10.1371/journal.pone.0026950)
Supplement: Table S3 — Sequences of semi-quantitative RT-PCR primers used to sequence and/or verify the size of mouse Topaz1 cDNA. (PDF) [file pone.0026950.s005.pdf]

Table S3 - Sequences of semi-quantitative RT-PCR primers used to sequence and/or verify the size of mouse *Topaz1* cDNA (mu, mouse)

Gene	Primers	size
mu*Topaz1* Exon 1	5′-GCACCACCCGAGGCCTGAGG-3′<br>5′-ACCTTCTGTCTCTTCTCTCC-3′	155bp
mu*Topaz1* Exon 2 A	5′-GAGGCCCCAAGTGATGATCC-3′<br>5′-GCAAGATTCCTAAGGTTTTC-3′	467bp
mu*Topaz1* Exon 2 B	5′-GACATCAATAACCAGTTCGG-3′<br>5′-TTCTCCCAGTGTTGTTCTCC-3′	593bp
mu*Topaz1* Exon 2 C	5′-AATAGCCAGTAAGTTACTGC-3′<br>5′-TTTTAACCAAGTCCGGTGCC-3′	1269bp
mu*Topaz1* Exon 2-4	5′-TAATGCAACTGAAGACACCC-3′<br>5′-TTCACTGAGGCCTTCTAGGC-3′	1087bp
mu*Topaz1* Exon 4-14	5′-ATGATCCTGAAGGAGCAGCC-3′<br>5′-AGCGTCTCAGCAACACAAGC-3′	1129bp
mu*Topaz1* Exon 14-20	5′-CTGGACCAAATTGGGAAACC-3′<br>5′-ATATTCGAGCAGCCATGACC-3′	1050bp
mu*Topaz1* Exon 20	5′-ACAGGTGCTGCAGATTGTCC-3′<br>5′-CCCTTTACTAATGGTTAGTG-3′	255bp
