# Supplementary material for: TOPAZ1, a Novel Germ Cell-Specific Expressed Gene Conserved during Evolution across Vertebrates
Source: PLoS One. 2011 Nov 1;6(11):e26950. doi: 10.1371/journal.pone.0026950 (PMC3206057; doi:10.1371/journal.pone.0026950)
Supplement: Table S4 — Primer sequences and conditions used for RT-PCR analyses. (PDF) [file pone.0026950.s006.pdf]

Table S4 - Primer sequences and conditions used for RT-PCR analyses (mu, mouse; ov, ovin)

Gene	Primers	Annealing temperature (°C)	MgCl2 (mM)	Cycles
**ov*TOPAZ1***	5′-GCTAGTAGTATTCAGAGTCC-3′<br>5′-TCAGGGCAGAACAATGTTCC-3′	56	2,5	30
**mu*Topaz1***	5′-AAAAGTGGGAGCCTAGATGG-3′<br>5′-TAGCAACCCAGTGAAAGAGC-3′	57	1,5	30
**ov*GAPDH***	5'-AGGCCATCACCATCTTCCAG-3'<br>5'-GGCGTGGACAGTGGTCATAA-3'	58	1,5	22
**mu*Actb***	5′-CATCCGTAAAGACCTCTATGCC-3′<br>5′-TAGAGCCACCAATCCACACAG-3′	55	2,5	28
