# Supplementary material for: TOPAZ1, a Novel Germ Cell-Specific Expressed Gene Conserved during Evolution across Vertebrates
Source: PLoS One. 2011 Nov 1;6(11):e26950. doi: 10.1371/journal.pone.0026950 (PMC3206057; doi:10.1371/journal.pone.0026950)
Supplement: Table S5 — Sequences of qPCR primers. Oligonucleotides for qPCR were designed using the PrimerExpress Designer software (Perkin Elmer). (PDF) [file pone.0026950.s007.pdf]

Table S5 - Sequences of qPCR primers (mu, mouse; ov, ovin)

Gene	Primers
ov*TOPAZ1*	5′-TTCATTAAGCACATGACCGTCAA-3′<br>5′-CCGGCCCACTTCATATTCTCT-3′
ov*DMC1*	5′ TCATACCCTCTGTGTGACAGCTC-3′<br>5′-GGCGATCTGGACGGAAAGTA -3′
ov*HPRT1*	5'-TGGACTAATTATGGACAGGACCG-3'<br>5'-TATAGCCCCCCTTGAGCACA-3'
mu*Topaz1*	5′-ACATTGCGTGGCTGTGAGC-3′<br>5′-CTCAGGCACATGGACAAACTTG-3′
mu-*Mvh*	5′-GAAGAAATCCAGAGGTTGGC-3′<br>5′-GAAGGATCGTCTGCTGAACA-3′
mu-*Actb*	5′-GCCCTGAGGCTCTTTTCCAG-3′<br>5′-TGCCACAGGATTCCATACCC-3′
mu-*Rec8*	5′-CCAAGGCCTGAACTCTCTTC-3′<br>5′-ATAGAAGACCCGAGAAGCCA-3′
mu*Wt1*	5′-GGCAGGAAAGTG TGCAAACTGCT-3′<br>5′-TGTGCTGTCTTG GAAGTCGGATGT-3′
mu*Hprt1*	5′-CAAAGCCTAAGATGAGCGCAA-3′<br>5′-AGGCAGATGGCCACAGGAC-3′
